# Chemical Cleaning Process of Polymeric Nanofibrous Membranes

**DOI:** 10.3390/polym14061102

**Published:** 2022-03-09

**Authors:** Aysegul Gul, Jakub Hruza, Lukas Dvorak, Fatma Yalcinkaya

**Affiliations:** Institute for Nanomaterials, Advanced Technology and Innovation, Technical University of Liberec, Studentska 1402/2, 46117 Liberec, Czech Republic; aysegul.gul@tul.cz (A.G.); jakub.hruza@tul.cz (J.H.); lukas.dvorak@tul.cz (L.D.)

**Keywords:** nanofiber, PAN, membrane, microfiltration, cleaning, chemical agents

## Abstract

Membrane fouling is one of the most significant issues to overcome in membrane-based technologies as it causes a decrease in the membrane flux and increases operational costs. This study investigates the effect of common chemical cleaning agents on polymeric nanofibrous membranes (PNM) prepared by polyvinylidene fluoride (PVDF), polyacrylonitrile (PAN), and polyamide 6 (PA6) nanofibers. Common alkaline and acid membrane cleaners were selected as the chemical cleaning agents. Membrane surface morphology was investigated. The PAN PNM were selected and fouled by engine oil and then cleaned by the different chemical cleaning agents at various ratios. The SEM results indicated that the use of chemical agents had some effects on the surface of the nanofibrous membranes. Moreover, alkaline cleaning of the fouled membrane using the Triton X 100 surfactant showed a two to five times higher flux recovery than without using a surfactant. Among the tested chemical agents, the highest flux recovery rate was obtained by a binary solution of 5% sodium hydroxide + Triton for alkaline cleaning, and an individual solution of 1% citric acid for acidic cleaning. The results presented here provide one of the first investigations into the chemical cleaning of nanofiber membranes.

## 1. Introduction

Today’s rapid urbanization and industrialization cause a rapid depletion of limited resources. Water is one of the most valuable resources on Earth, but has come seriously under threat from contaminants due to undesirable human activities such as marine dumping, as well as domestic, industrial, and agricultural practices.

Approximately 40% of the world’s population lives in areas with water issues. Although 70% of the earth is covered with water, the proportion of freshwater is low. Only 3% of the water on the planet is considered suitable for human consumption. It is known that 1.2 billion people do not have access to clean drinking water; however, this number may reach 3.5 billion by 2025 [1].

Taking this into consideration, one of the biggest challenges today is the development of highly efficient and cost-effective water treatment technologies. The most widely used water treatment technologies today are pressure-driven membrane filtration processes (including microfiltration (MF), ultra-filtration (UF), nanofiltration (NF), and reverse osmosis (RO)). These systems have certain advantages and disadvantages and are open to development. For example, while the thermal stability and durability of ceramic MF are relatively good, they have lower permeability and higher costs than other processes [2].

Nowadays, researchers are focusing more on studying how nanotechnology may be integrated into membrane systems to improve membrane stability, where water treatment is gaining tremendous importance. The most common techniques used in nanofiber production are two-component extrusion, phase separation, template synthesis, drawing, melt blowing, electrospinning, and centrifugal spinning [3,4]. Of these, electrospinning attracts more attention from researchers due to its unique properties, for example, high porosity and large surface area, etc.

Electrospun nanofibers have specific advantages such as high porosity, tight pore size, and a large surface area. These properties make them unique candidates for water treatment applications [5]. The largest obstacle for the commercialization of electrospun nanofibrous membranes is their low mechanical properties, which is why research is generally focused on improving these properties.

Yalcinkaya et al. [6] stated that the mechanical strength of nanofibers may be increased by using a support layer via a lamination technique. Makaremi et al. [7] developed electrospun PAN nanofibrous membranes with improved mechanical properties, thermal stability, and water filtration performance, using 1%, 2%, and 3% *w*/*w* halloysite nanotubes (HNTs) for applications such as water filtration membranes. Wirth et al. [8] used ultrasonic welding to combine nanofiber webs on a support, which has potential application in the field of water treatment.

Electrospun nanofiber membranes are suitable for use in microfiltration. In a previous study [9], hydrophilic/oleophobic microfiltration membranes were produced using wire electrospinning for the separation of oil–water emulsions. Polyvinylidene fluoride (PVDF), polyacrylonitrile (PAN), and PVDF/PAN nanofiber hybrid membranes were prepared. The best water permeability was observed for the neat PAN nanofibrous membrane, while PVDF facilitated oil permeability. The results showed that nanofiber membranes are excellent for the separation of emulsions. Ma et al. [10] studied functionalized electrospun nanofibrous microfiltration membranes for the removal of bacteria and viruses from contaminated wastewater. They used a modification method to improve the mechanical properties of PAN electrospun nanofibrous membranes. The results indicated that the developed electrospun PAN nanofibers provided 99.99% bacteriophage retention during microfiltration compared to commercial microfiltration membranes. Lee et al. [11] studied fouling-tolerant nanofibrous polymer membranes for water treatment. They developed electrostatically negatively charged Nafion/PVDF nanofibrous membranes on the fiber surface, with superior water permeability and anti-fouling behavior compared to conventional microfiltration membranes produced by the electrospinning method.

Even though nanofibrous membranes may be beneficial when used in membrane technologies, membrane fouling is an inevitable phenomenon during filtration. Membrane fouling affects the membrane’s lifespan at the membrane surface or pores. When the process is run under constant transmembrane pressure (TMP) or constant flux conditions, membrane fouling causes a significant increase in hydraulic resistance, manifested as a decrease in permeate flux or an increase in TMP. The energy required to achieve filtration increases in a system where the permeate flux is maintained by increasing TMP. Fouling resulting from long-term filtration is not entirely removed by backwashing [12].

Fouling causes a deterioration of membrane performance, restricts the efficient operation of membranes, and increases operational costs. Factors affecting fouling are membrane properties, feed solution properties, and operating conditions. Membrane properties are pore size, hydrophobicity, pore size distribution, and membrane material. Solution properties are solid (particle) concentration, particle size, and nature of components. Operating conditions are pH, temperature, flow rate, and pressure [12].

Typically, foulants may be divided into four categories: particulates, organic, inorganic, and microbiological organisms. Depending on the bonding strength of the foulants to the membrane surface, membrane fouling is divided into two classes: reversible fouling and irreversible fouling. Foulants that cause reversible fouling are inorganic compounds, organic compounds, microorganisms, bacteria, and their metabolites. Reversible fouling may be removed by the effective shear force of backwashing or relaxation under cross-flow conditions, called physical cleaning [13,14]. Physical cleaning is not adequate for the removal of irreversible fouling. Irreversible fouling is considered as permanent fouling, and it is not removed by any cleaning method without chemical agents. In this situation, membrane chemical cleaning is necessary to maintain the permeability and selectivity of a membrane process.

Membrane cleaning helps to recover membrane fouling and to extend its flux, selectivity, and lifespan. During chemical cleaning, a reaction occurs between the pollutants on the membrane surface and the chemical agents. The cleaning agent either cleans the membrane by removing foulants while changing the morphology of the foulants or changes the surface chemistry of the fouled layer.

Chemical cleaning agents may be divided into the following seven categories: caustics (e.g., sodium hydroxide), alkalis (e.g., carbonates, hydroxides, phosphates), acids (e.g., nitric acid, sulfuric acid, phosphoric acid, citric acid, oxalic acid), enzymes (e.g., proteases and lipases), surfactants (e.g., alkyl sulfate, sodium dodecyl sulfate, cetrimonium bromide), sequestrants (e.g., ethylenediaminetetraacetic acid), and disinfectants (e.g., metabisulphite, sodium hypochlorite, peroxyacetic acid, hydrogen peroxide, chlorine, and hypochlorite) [15]. In addition to these main categories, a blend of various cleaning agents or a combination with other physical cleaning is also commonly adopted.

The type of chemical agent used is determined based on the structure of the membrane and the foulant (i.e., proteins, glucans, pigments, minerals, hydrophobes, starch, tannins, pectin, and fat). Chemical agents must be safe, cheap, and washable with water, also they must be able to dissolve most of the precipitated foulants on the surface and should not damage the membrane surface while removing foulants [16]. The interactions that may occur between chemical agents and contaminated membranes may be listed as follows: hydrolysis, peptization, saponification, solubilization, dispersion (suspension), and chelation [17]. In addition to the types of chemical agents, other factors affecting the chemical cleaning efficiency include cleaning time, concentration, cleaning temperature, etc.

Chemical cleaning may negatively affect the physical and chemical properties of the membrane, causing damage to the membrane surface and deterioration of selectivity performance. This study focused on two stages: the first stage focused on the effect of the cleaning agent on the surface morphology of various nanofibrous membranes prepared from PVDF, PAN, and polyamide 6 (PA6) nanofiber webs. Various different alkaline (e.g., sodium hydroxide (NaOH)) and acid (e.g., citric acid (CA)) cleaning agent concentrations were used. The nanofibrous membranes were treated with chemical agents and each sample was kept in the prepared solutions for 24 h. The types of chemical agents and process conditions were the concentration of the cleaning solution (except surfactants). The second stage of the work is related to the effect of the chemical cleaning agents on flux recovery of the nanofibrous membrane. In the second part of the study, PAN nanofibrous membranes were tested under a cross-flow module to separate oily wastewater. Four types of chemical cleaning agents were selected for the chemical cleaning of the fouled nanofibrous membranes: alkaline (NaOH), acid (CA), and surfactants (sodium dodecyl sulfate (SDS) and Triton). The effect of the combination of chemicals was tested with cleaning sequences of alkaline, acid, and alkaline–acid-surfactants.

Membrane cleaning is important to reduce fouling and enhance its permeability, followed by performance, in a membrane-based treatment plant. Membrane fouling can be minimized by membrane cleaning. Generally, cleaning protocols of membranes are recommended by the membrane manufacturers. To date, no work has been reported in the literature for the chemical cleaning of nanofibrous membranes. This work is a priority for future investigations of the chemical cleaning of membranes prepared by nanofiber webs. Today, developing a suitable chemical cleaning process that uses fewer chemicals and less energy is a step forward for the green economy. As a matter of fact, it is also worth noting that in this study, the use of relatively mild cleaning conditions, such as shorter cleaning times and lower chemical concentrations, was critical.

## 2. Materials and Methods

### 2.1. Preparation of Membranes and Chemical Cleaning Agents

Polymeric nanofiber membranes (PNM) were prepared using a lamination method. The electrospinning method was used for the preparation of the nanofiber layers. The Nano-spider electrospinning device was used at CXI, TUL (Technical University of Liberec, Liberec, Czech Republic). Nanofibers were cut into A4 size (210 × 297 mm^2^). A PET spunbond, nonwoven (Mogul Nonwovens, Gaziantep, Turkey), was used as a supporting layer, and an adhesive web was used to adhere the supporting spunbond layer and the nanofiber layer. The lamination process was performed using heat-press equipment (Pracovni Stroje, Teplice, Czech Republic). The adhesive layer was placed between the nanofiber layer and the spunbond support layer. The prepared sandwich structure was placed in heat-press equipment with upper and lower plates. Two silicone layers were used between the hot plates and the nanofibers to prevent the nanofibers from contacting directly with the hot plates. Heat was applied at a temperature of 130 °C for 3 min under a force of 50 kN. The commercial polyethylene terephthalate (PET) spunbond, nonwoven, was supplied from Mogul (Mogul, Gaziantep, Turkey). Various nanofibers were taken from CXI, TUL (Technical University of Liberec, Liberec, Czech Republic) at the same fiber densities (g/m^2^as gsm). Table 1 shows the materials used in the lamination. Various chemicals were used as cleaning agents, as shown in Table 2. All of the chemical cleaning agents were purchased from Sigma-Aldrich (Sigma-Aldrich s.r.o., Prague, Czech Republic).

In the first stage (Stage 1) of the experiment, the PA6, PAN, and PVDF PNMs were immersed into two different concentrations (1 and 5 wt.%) of NaOH and CA, and only 2 wt.% of surfactant triton and SDS during 24 h. The aim was to investigate the effect of selected alkaline and acidic chemical cleaning agents on nanofiber membrane surface morphology.

In the second stage (Stage 2) of the experiment, PAN PNM was selected for the cleaning process. The membranes were contaminated using wastewater from an engine oil/water 50/50 *v*/*v* mixture. NaOH, CA, and surfactant (triton and SDS) were used as cleaning agents. The concentrations of NaOH and CA were 1 and 5 wt.%, while surfactants were 2 wt.%, which was determined according to previous studies related to the membrane cleaning process [18]. Later, the mixtures of surfactants with various concentrations of NaOH and CA were prepared for cleaning purposes. The membrane flux recovery rates before fouling and after cleaning of fouled membranes were compared.

Table 3 shows the amount of chemical cleaning agents used during the first and second stages of the experiment.

### 2.2. Evaluation of the Cleaning Agent’s Effect on the Membrane Surface

The nanofibrous membranes were treated with various chemical cleaning agents at two different concentrations for 24 h in the first stage of the research. The effect of the chemicals on the morphology membrane surface was examined using a scanning electron microscope (Vega, 3SB, Brno, Czech Republic). A Nicolet iZ10 Fourier transform infrared spectroscope (FTIR; Thermo Scientific, Prague, Czech Republic) was used to characterize changes in the chemical structure of the membrane after the chemical cleaning process. The types and amounts of chemicals are illustrated in Table 2. The amounts of chemical cleaners were selected according to the literature [18].

The surface contact angle of the samples with deionized water (surface tension 72.0 mN m^−1^) was measured at five different points on clean and dry samples at room temperature using a Krüss Drop Shape Analyzer DS4 (Krüss GmbH, Hamburg, Germany). The pore size of the membranes was determined by the bubble point method using a Porometer 3G through a pore size analyzer (Quantachrome Instruments, Anton Paar GmbH, Germany). The tests were performed according to the ASTM F316-03 (2011) standard.

### 2.3. Filtration Test

**Figure 1:**
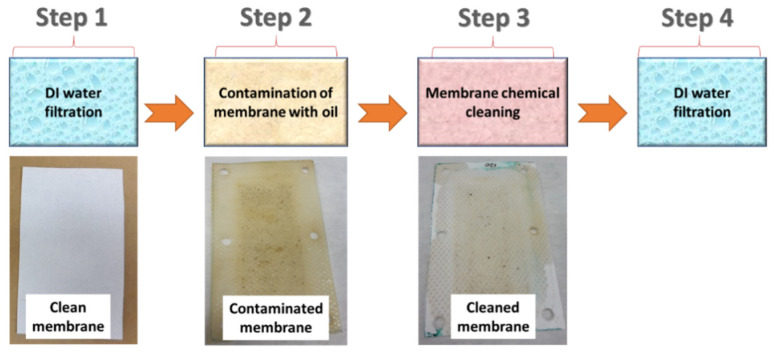
Stage 2 (the membrane chemical cleaning process).

A custom-made cross-flow module was used for the oily wastewater separation. The flow rate of the feed was stable at 15 L/min. Figure 1 shows the experimental steps for the chemical cleaning process (Stage 2). A highly concentrated engine oil/water mixture (oil properties and the company name were not provided by the supplier) was used as a feed solution for the fouling of the membranes. Firstly, distilled water (DI water) was used as the feed for 60 min and its permeability was recorded (Step 1). Secondly, the membranes were exposed to oily wastewater for 180 min (Step 2). If the membrane permeability did not decrease to zero, then Step 2 was continued until the permeability stopped. After the membranes were totally blocked, a chemical cleaning agent added to the DI water was used as the feed for 10 min (Step 3). The DI water was used as the feed for the cleaned membranes for 60 min and their permeability was recorded (Step 4).

Membrane permeability (*P*) and flux recovery rate (*FRR*) were evaluated using Equations (1) and (2):

(1) $$P=\frac{L}{Atk}\;({\mathrm{Lm}}^{-2}h^{-1}{\mathrm{bar}}^{-1})$$

*FRR* (%) = (*P*1*/P*0) 100 (2)

where *L* is the amount of permeate (liter), *A* is the area of active membrane (m^2^), *t* is the duration of the filtration test (h), *k* is the transmembrane pressure (bar), *P*0 is the initial DI water permeability at Step 1, and *P*1 is the DI water permeability at Step 4. The pH values of the prepared chemical cleaning solutions are presented in Table 4.

## 3. Results

### 3.1. Cleaning Agent’s Effect on the Membrane Surface

The effects of the chemicals used on the surface morphology were investigated by SEM, FTIR, and pore size analyses. SEM images of the membranes before and after contact with the chemical cleaning agents are provided in Table 5, Table 6 and Table 7 for Stage 1. The aim of this test was to observe if any of selected chemicals at selected concentrations could cause damage on the nanofiber surface.

A low concentration of CA in particular may be used in PAN membrane cleaning systems without causing significant damage to the membrane [19]. CA provides buffering and has good chelating abilities, making it effective and simple to use with a lower risk of pH damage to the membrane [19]. Herein, CA did not damage any of the PNMs.

A high concentration of NaOH caused swelling of fibers and reduced the porosity of the PAN membrane. Studies in the literature support our findings. Bryjak et al. [20] stated in their study that NaOH-induced hydrolysis of nitrile groups on the surface of PAN membranes resulted in a decrease in pore diameter due to swelling of the membrane. Oh et al. [21] also examined the hydrolysis of PAN membranes by changing the NaOH solution concentration and duration, and by keeping the temperature constant. They observed that at lower NaOH concentrations, morphological changes were negligible, and changes in hydrophilicity were dominant. They determined that at higher NaOH concentrations, morphological changes became more pronounced with increased hydrophilicity. In this work, low concentrations of NaOH were used. No damage was observed on PNMs.

Rabuni et al. [22] mentioned in their study that alkaline cleaning (NaOH) affected the surface properties of PVDF membranes such as wettability. Pristine PVDF is naturally hydrophobic. PVDF may be converted into a hydrophilic membrane by modifying its surface using alkaline treatment [23,24]. It was found that PVDF membranes can be attacked and degraded upon exposure, even to a low concentration of NaOH (0.01 M) solution [25]. In our case, both SEM and FTIR results did not show any changes on the PVDF PNM.

It was found that an alkali-catalyzed hydrolysis PA membrane was observed after post-treatment at pH 14 for 28 days [26]. Herein, PA6 PNM was kept in NaOH for 24 h, which did not show any changes.

**Figure 2:**
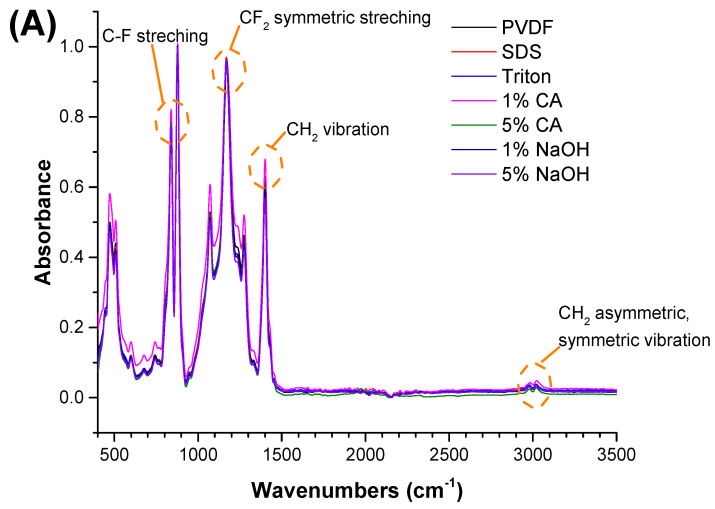

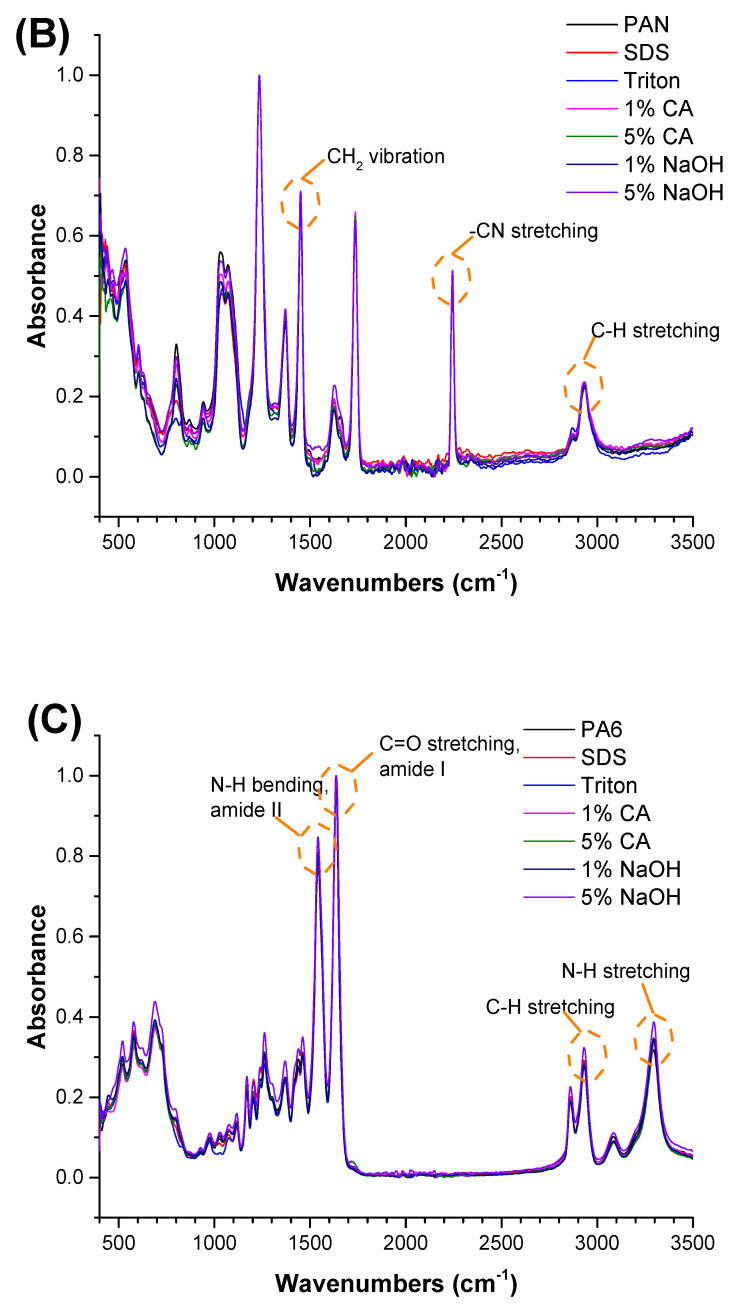
FTIR spectra of the (**A**) PVDF, (**B**) PAN, and (**C**) PA6 membranes before and after the chemical cleaning process.

Clearly, none of the chemical cleaning agents showed any damage on the membrane surface after 24 h of immersion. The surfactant effects were observed under SEM and FTIR (Figure 2). Surfactants did not affect the PNMs.

The FTIR test was performed to understand any chemical changes after contact with the chemical cleaning agent. Membranes were cleaned with distilled water after contact with the chemical cleaning agent and dried before FTIR. The FTIR results indicated that there were no significant changes after the membranes came into contact with the chemical cleaning agents. The characteristic peaks for each polymeric membrane were observed, and are shown in Figure 2.

**Figure 3:**
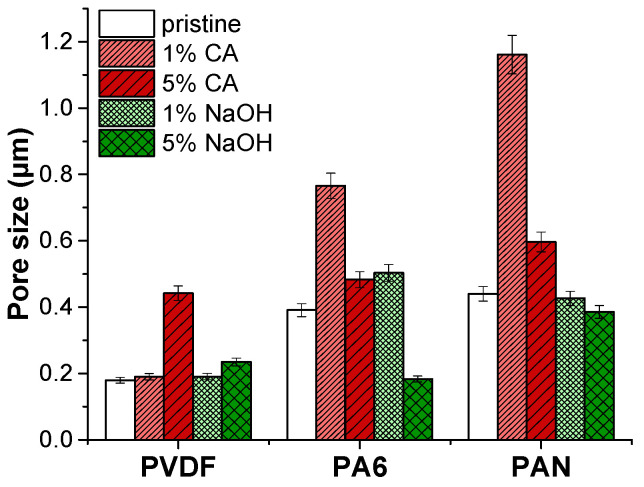
Pore size of membranes after the application of chemical cleaning agents.

Pore size results of the pristine membranes and membranes after the application of the chemical cleaning agents are shown in Table 8 and Figure 3.

The average pore size of PVDF membranes increased slightly under the 5% NaOH cleaning agent. It was found that by increasing the concentration of NaOH and the treatment temperature, the mechanical integrity of the PVDF membranes was destroyed entirely in the 4 wt.% NaOH solution at 70 °C as a result of a decrease in elongation and crystallinity [27]. Similarly, increasing the NaOH concentration may cause deterioration of PVDF, which results in an increased pore size. However, the pore size of the PVDF membrane remained intact after the CA chemical cleaning agent.

The PA6 membrane’s pore size increased under a low alkaline (caustic) chemical cleaning agent (1% NaOH) concentration. It was found that NaOH cleaning agents cause a slight increase in the average pore size of the NF270 membrane [28]. On the other hand, increasing the concentration of NaOH up to 5% caused a 2-fold reduction of the PA6 membrane’s pore size. Low concentrations of the CA cleaning agent increased the pore diameter by almost 2-folds compared to the pristine PA6 membrane. At a high concentration of the CA cleaning agent, the pore size of the PA6 membrane slightly increased. Under extreme pH conditions, the structure of amide bonds in polyamide can be easily destroyed by an acid or base. Since hydrogen or hydroxide ions react with oxygen/nitrogen or carbon atoms in amide bonding, this can abate the resonance structure of an amide and cause hydrolysis of the amide bond [26]. Moreover, under strong acidic and alkaline conditions, the charged groups in the membrane matrix can cause internal charged repulsion, resulting in expansion of the membrane structure and pores.

In the case of PAN membranes, under alkaline cleaning, the PAN fibers are swollen, and the pores are reduced. Similar results have been observed before [20,29]. It was observed that NaOH treatment exhibited swelling of PAN membranes, resulting in a pore diameter reduction [30,31]. The NaOH cleaning agent caused hydrolysis of nitrile groups on the membrane surface. As a result, the membrane swells, pore size reduces, and the membrane surface becomes smooth.

On the contrary, the acidic cleaning agent CA caused a pore size increment. It was stated that PAN membranes did not lose performance with the use of CA chemical cleaning [19]. At concentrated acids, PAN starts hydrolysis [32]. FTIR results indicated that there is no hydrolysis of PAN membranes. In this case, some nanofiber layer deformation might occur in the CA chemical cleaning process, increasing the average pore diameter.

### 3.2. Results of Flux Recovery after Chemical Cleaning

#### 3.2.1. Contact Angle Results

The hydrophilicity of the PAN membrane surface was investigated by measuring the contact angle of water using the sessile drop method on membranes cleaned with chemical agents. Fouling of membranes and chemical cleaning are recognized to significantly change the properties of the membrane surface, such as roughness and hydrophobicity. One of the main surface properties analyzed is the contact angle [33]. The lower the contact angle, the more hydrophilic the membrane. This means that when the other membrane characteristics such as pore size and pore density are the same, the lowest contact angle yields the highest membrane permeability. PAN is inherently hydrophilic [34,35]. Table 9 shows the contact angle results. The average contact angle of a pristine membrane was measured as 81.44 (±3.44). The contact angles became hydrophilic due to alkaline cleaning and, similarly, the use of Triton caused a decrease in the contact angle.

PAN is hydrolyzed in alkaline conditions, and nitrile groups on the PAN membrane surface turn into functional carboxylate groups. PAN hydrolysis has been employed in the preparation of highly hydrophilic membranes [31,36]. Increasing the NaOH concentration yielded a decrease in the contact angle of PAN PNM. The citric acid did not significantly influence the membrane water contact angle.

Citrate-based cleaning can enhance the emulsifying properties of the SDS-based micellar system. It was found that SDS surfactant inactivation increased with the salt concentration [37]. A more closely packed monolayer was formed in the mixed-additive surfactant compared to individual SDS molecules. The reason was the attractive electrostatic, interfacial adsorption, or hydrophobic interactions between the two species. The water contact angle of the membrane increased after 5 wt.% CA + SDS and 5 wt.% NaOH + SDS treatment. The interaction between NaOH-SDS and CA-SDS would cause changes in the membrane surface charge, which reduces membrane wettability. Moreover, SDS has a hydrophobic 12-carbon chain and a polar sulfate head group [38]. The hydrophobic part of SDS is placed on the membrane surface, while the hydrophilic part interacts with CA and the membrane. As a result, the water contact angle is increased.

#### 3.2.2. Alkaline Cleaning

**Figure 4:**
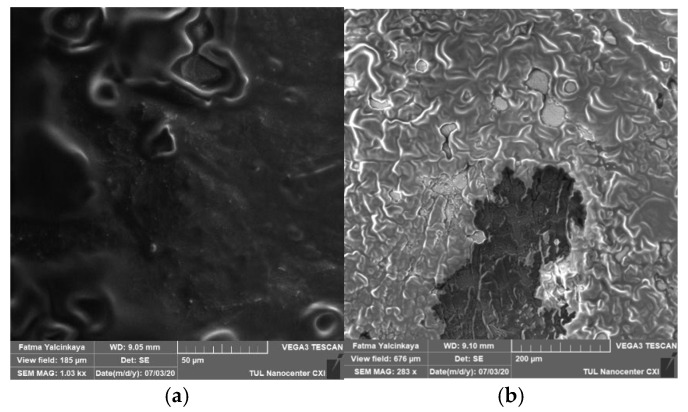
Top view of a membrane fouled by engine oil (**a**), and an image of the fouled membrane after the chemical cleaning process (**b**).

Alkaline cleaning agents may remove organic fouling by increasing negative charges on both solutes and membranes, dilating membrane pores, protein hydrolysis, or dissolving foulants, and they may even dissolve silicon salts that are insoluble in acids [18]. Alkaline cleaning (NaOH) was investigated based on the concentration and the effect of surfactants. Figure 4 shows a top view of a membrane fouled by engine oil (a), and an image of the fouled membrane after the chemical cleaning process (b).

##### Effect of Concentration

**Figure 5:**
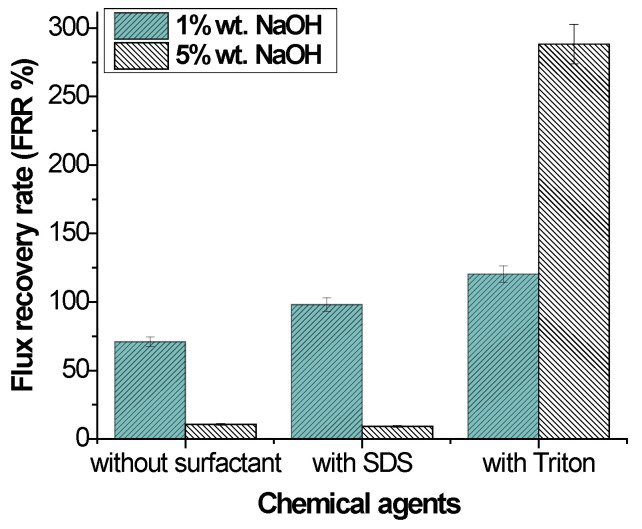
Effect of chemical agents on flux recovery by alkaline cleaning.

The relationship between the concentration, contact angle, and flux recovery rate was investigated. NaOH was used as an alkaline cleaning agent. The flux recovery rate is shown in Figure 5. The lowest concentration of the NaOH solution without surfactant showed the highest flux recovery rate. With an increase in the NaOH concentration from 1% to 5%, the hydrophilicity of the membrane increased, as shown in Table 9, but the flux recovery rate and permeability decreased. A high alkaline concentration may cause membrane swelling, leading to a reduction in pore structure, as shown in Figure 3.

Sodium hydroxide acts in two ways: (1) hydrolysis and (2) solubilization. Moreover, fats and oils react with caustic substances through saponification. This process generates water-soluble soap micelles [39]. Some studies mention that the NaOH-induced hydrolysis of nitrile groups on the membrane surface causes a reduction in both pore diameter and membrane permeability [20,40,41]. According to certain studies, morphological changes may be overlooked at low NaOH concentrations, and they become more pronounced as NaOH concentrations increase [30].

Adding a surfactant to an alkaline cleaning agent improved the membrane FRR. The reason could be due to the adsorption of surfactants onto the surface of PAN PNM. Adsorbed surfactants can greatly modify membrane permeability to water. It was proposed that, when the surfactant is adsorbed to the body of certain membranes, it can modify the membrane permeability [42].

The alkaline cleaning agent together with the surfactant decreased the fouling due to the reduced interaction between the oil pollutant and the membrane surface and the negatively charged PAN PNM at a high pH (between 10 and 13). At a high pH range, the carboxyl groups on PAN PNM would be deprotonated and produce a negative charge on the membrane surfaces. It was found that the membrane negative charge increased with the increased pH value [43].

##### Effect of Surfactants

The flux recovery using binary solutions of SDS + NaOH and NaOH + Triton is shown in Figure 5. Both surfactants were effective for flux recovery. The highest value of cleaning efficiency was achieved using Triton as the surfactant, with a flux recovery of 288.84% at a 5% concentration of NaOH. While alkaline treatment alone was sufficient to promote super-hydrophilic functionality, previous research has shown that surfactants also improve membrane surface hydrophilicity [31]. Triton is a non-ionic surfactant and may be responsible for increasing the membrane’s affinity for water.

SDS showed a positive effect on flux recovery, 98.12% when the concentration of NaOH was the lowest. SDS is an anionic surfactant. The effect of SDS (surfactant) is linked to the cleaning strength of emulsifiers as a result of changing the water’s interfacial tension [44]. The synergistic effects of 5% NaOH and SDS on the membrane surface may be attributable to this outcome, where adsorption of SDS on the membrane surface may have caused pore clogging [45,46,47].

#### 3.2.3. Acidic Cleaning

Citric acid, hydrochloric acid, sulfuric acid, and other acidic cleaning agents are frequently used to remove inorganic foulants by weakening the interaction of foulants and membranes and dissolving the deposited inorganic salts or metal oxides [18]. Acidic cleaning was investigated based on the concentration and the effect of surfactants.

##### Effect of Concentration

**Figure 6:**
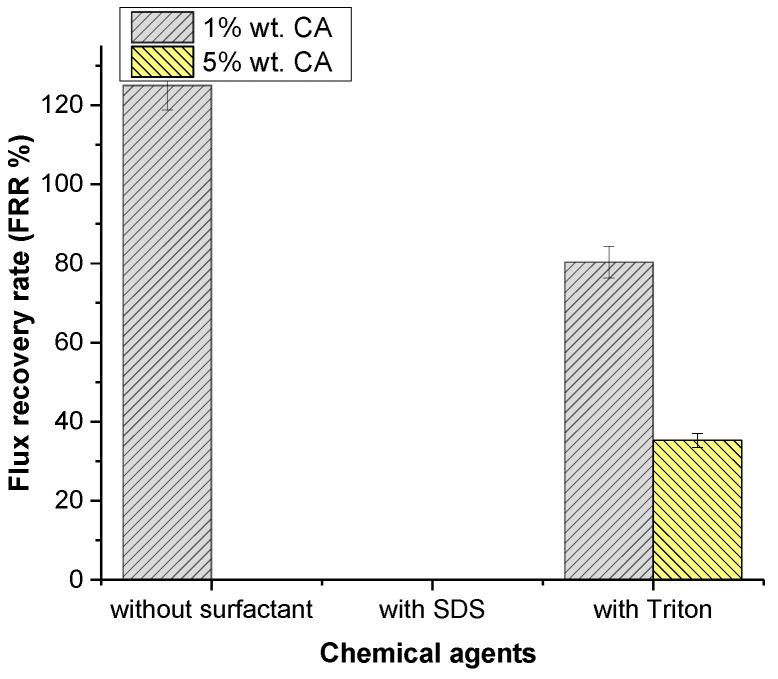
Effect of chemical agents on flux recovery by acidic cleaning.

Citric acid was used as an acidic cleaning agent. The flux recovery is shown in Figure 6. The lowest concentration of citric acid showed an enormous effect on flux recovery of 125.01%. Table 4 shows that the concentration increases the acidity of the solution, which may influence the cleaning of the membrane. Table 4 and Table 9 show that increasing the concentration of CA from 1% to 5% slightly decreased the chemical cleaning solution pH and the hydrophilicity of the membrane surface, which resulted in a negative effect on flux recovery.

Generally, organic foulants contain carboxylic acid groups. Under acidic conditions, these groups contain a hydrogen ion, which does not include a net charge on the molecule. On the contrary, at a high pH, the carboxylic acid groups dissociate and leave a negative net charge on the molecule, which results in higher hydrophilicity. Acidic chemical cleaners with low pH values are more suitable for inorganic fouling compounds than for organic ones.

The highest flux recovery (125.01%) was observed at the lowest concentration of CA. Citric acid contains hydrophilic monomers, which may increase the membrane surface’s hydrophilicity [48]. Table 9 shows the changing hydrophilicity of the membrane, which supports our findings for 1% CA. However, this was only effective up to a certain concentration, and the presence of high concentrations of CA hampered flux recovery. Table 4 shows that increases in the concentration of CA decreased the pH of the solution, which may lead to a decrease in flux for higher concentrations of CA.

At a low pH, the functional carboxyl groups of PAN PNM were protonated and caused a neutral charge on the membrane surface [49]. As a result, membrane fouling increased.

##### Effect of Surfactants

The effect of various surfactants on cleaning membranes was investigated and the results are shown in Figure 6. When SDS was compared to Triton, Triton had an undeniable effect on flux recovery, with 80.81%. Contrary to the literature, Triton improved the hydrophilicity of the membrane’s surface at low CA concentrations, but it had less influence on the flux increase compared to individual CA cleaning agents. This issue may be related to an increase in roughness. SDS was observed to completely stop flux recovery. When the contact angle results were examined, it was evident that SDS reduced the surface’s hydrophilicity. Once the SDS surfactant micellizes, SDS colloids may cause membrane fouling as colloidal deposition on the membrane surface results in membrane fouling [48]. There was a decrease in the flux as a result of fouling. Triton appears to considerably boost the membrane’s hydrophilicity, as can be seen in Table 9. Improvement was also seen in flux recovery as the hydrophilicity increased.

It was determined that PAN membranes showed instability against high concentrations of NaOH and resistance to CA and weak alkaline solutions [50]. CA alone seems to be a good chemical cleaner for PAN nanofibrous membranes.

It is possible to concluded that:

- CA as a chemical agent for cleaning PVDF, PA6, and PAN nanofibrous membranes will not harm the fibers. NaOH can causes swelling of the PAN membrane.
- The optimal concentration is 1 wt.% for both individual CA (acidic) cleaning and NaOH (alkaline) cleaning.
- A flux recovery rate of 288.84% was achieved through mixed cleaning with 5% NaOH and Triton, which was found to be better than individual cleaning.
- Individual CA cleaning had the greatest effect on flux recovery compared to individual NaOH cleaning.
- SDS had a negative effect on membrane flux in an acidic environment.

Fouling is one of the biggest problems of membrane technology. Extensive research is needed. A greater understanding of the various pollutants, the fouling mechanism on membrane types and modules, the cleaning mechanism, cleaning chemicals, and cleaning conditions is essential to improve membrane performance and minimize costs.

The type of used chemical is not the only parameter that affects chemical cleaning, but also factors such as time, temperature, and pH. Our future research will focus on investigating the impact of different temperatures on chemical cleaning. Temperature impacts on the chemical cleaning process and membrane flux have been discussed in the literature [18,51,52]. Temperature can affect chemical cleaning by altering the kinetics or balance of the reaction as well as the solubility of the pollutants. It has been demonstrated that increasing the temperature increases the reaction kinetics of the oxidation process, as well as the solubility of the foulants or reaction products and the distribution of reagents in a polymer matrix. Zhang et al. [53] demonstrated that increasing the temperature was advantageous to the recovery of membrane flux when performing chemical cleaning of a fouled membrane while treating wastewater from a banknote printing plant. Bartlett et al. [54] discovered that for two types of membranes, the recovery of membrane flux increased as the temperature increased from 30 to 50 °C under laboratory conditions.

This study contributes significantly to chemical cleaning research by demonstrating the surface morphologies and flux recovery rates of nanofibrous membranes. The findings can be useful in the cleaning process of nanofiber membranes in wastewater treatment. Future research will focus on the impacts of different temperature values on pollutant solubility, chemical reaction equilibrium, and reaction kinetics, as well as make broader contributions to the field.

## 4. Conclusions

The objective of this work was to observe the effect of chemical cleaning agents on nanofibrous membranes.

In summary:

- Nanofibrous PAN membranes were cleaned with various solutions in order to investigate the effect of concentration and chemical agents on cleaning efficiency. Clearly, determining the optimal concentration of cleaning agents is advantageous because high concentrations of cleaning agents are unfavorable in terms of cost and nanofibrous membrane integrity. The results showed that low concentrations for individual CA (acidic) cleaning and NaOH (alkaline) cleaning improved flux recovery, whereas increasing the concentrations decreased flux recovery. For both chemical agents, the optimal concentration was 1 wt.%.
- SDS showed the highest effect on flux recovery, with 98.12% at the lowest NaOH concentration. Binary solutions of SDS + NaOH were more effective than individual NaOH cleaning agents. The results also demonstrated that the highest flux recovery was achieved using a binary solution of Triton + 5% NaOH. Overall, recovery of flux reached a maximum of 288.84%. The addition of Triton to a NaOH solution proved to be an effective cleaning agent for organically fouled nanofibrous PAN membranes.
- Adding SDS to a CA solution during acidic cleaning created a flux stopper effect. Residual SDS deposited on the membrane surface may cause fouling of the membrane and limit favorable chemical reactions during chemical cleaning. The binary solution of Triton + CA provided a weaker flux recovery compared to alkaline cleaning.

This study makes a major contribution to research on chemical cleaning of nanofibrous membranes by demonstrating their surface morphology and flux recovery rates. The results will provide useful information for the process of cleaning nanofiber membranes in wastewater treatment.

## Figures and Tables

**Table 1:** Types of polymeric nanofibers and support layers.

Nanofiber	Spunbond Nonwoven
PVDF (2 gsm)	PET (100 gsm)
PAN (2 gsm)	PET (100 gsm)
PA6 (2 gsm)	PET (100 gsm)

**Table 2:** Types of cleaning agents.

Chemical	Type
Sodium hydroxide (NaOH)	Alkaline
Citric acid (CA)	Acid
Triton X 100	Surfactant
SDS	Surfactant

**Table 3:** The amount of cleaning agent used for the experimental stage 1 (chemical effects on the membrane surface) and stage 2 (the membrane chemical cleaning process).

	Amount of Chemical Cleaning Agent	
Type of Chemical Cleaner	Stage 1	Stage 2
NaOH	1 and 5 wt.% in DI water	1 and 5 wt.% in DI water
CA	1 and 5 wt.% in DI water	1 and 5 wt.% in DI water
SDS	2 wt.% in DI water-	2 wt.% SDS 2 wt.% SDS/1 wt.% CA 2 wt.% SDS/5 wt.% CA 2 wt.% SDS/1 wt.% NaOH 2 wt.% SDS/5 wt.% NaOH
Triton	2 wt.% in DI water	2 wt.% Triton 2 wt.% Triton/1 wt.% CA 2 wt.% Triton/5 wt.% CA 2 wt.% Triton/1 wt.% NaOH 2 wt.% Triton/5 wt.% NaOH

**Table 4:** pH values of chemical cleaning solutions.

Cleaning Solutions	Without Surfactant	With 2 wt.% Triton	With SDS 2 wt.%	2 wt.% Triton in Water	2 wt.% SDS in Water
1 wt.% NaOH	11	10	13	6.5	7
5 wt.% NaOH	12	12	13		
1 wt.% CA	2	2	2		
5 wt.% CA	1.5	1.5	1.5		

**Table 5:** SEM images of membranes after the application of chemical cleaning agents (1% CA-1% NaOH).

Polymers	Pristine Membrane	24 h	
		Chemical Agents	
		1% CA (1 wt.%)	1% NaOH (1 wt.%)
PA6	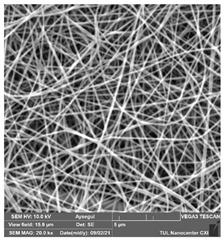	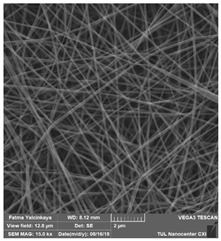	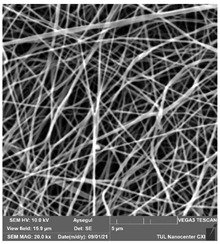
PAN	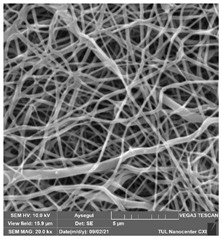	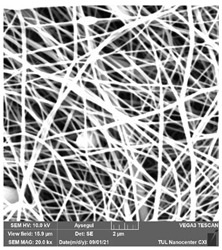	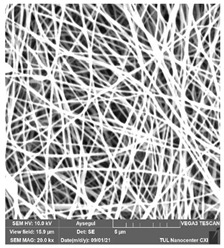
PVDF	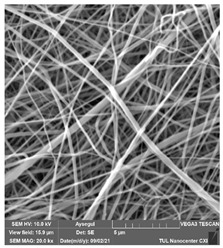	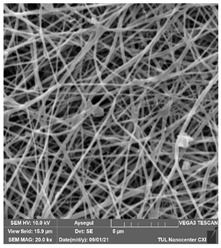	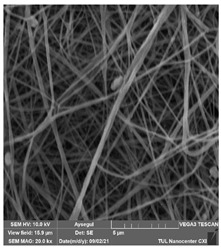

**Table 6:** SEM images of membranes after the application of chemical cleaning agents (5% CA-5% NaOH).

Polymers	Pristine Membrane	24 h	
		Chemical Agents	
		CA (5 wt.%)	NaOH (5 wt.%)
PA6	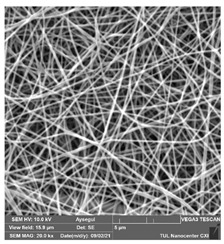	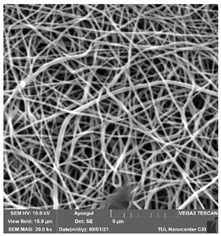	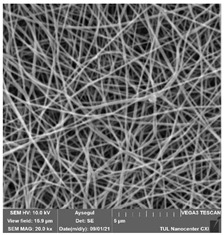
PAN	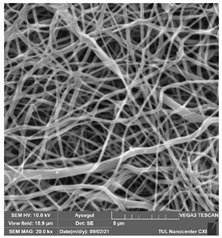	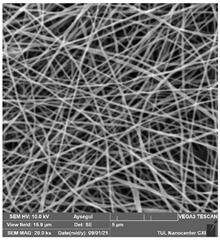	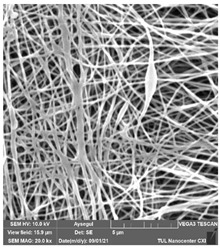
PVDF	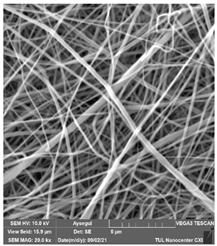	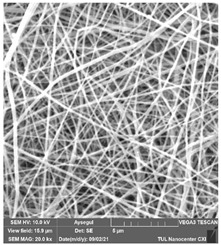	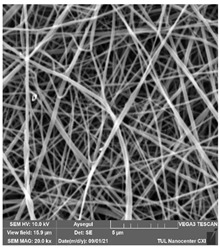

**Table 7:** SEM images of membranes after the application of chemical cleaning agents (2% Triton-2% SDS).

Polymers	Pristine Membrane	24 h	
		Chemical Agents	
		Triton (2 wt.%)	SDS (2 wt.%)
PA6	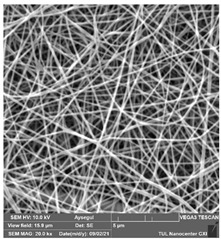	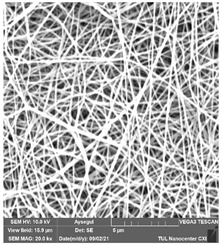	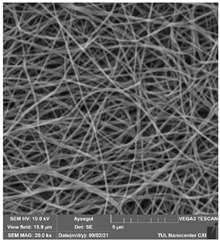
PAN	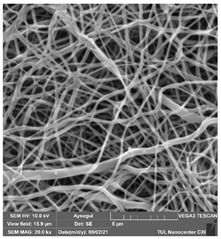	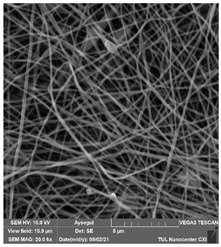	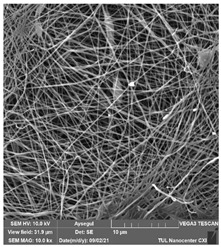
PVDF	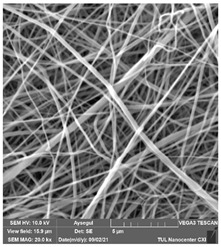	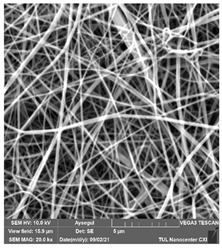	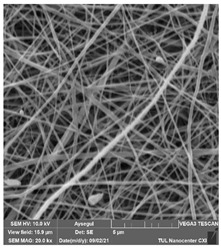

**Table 8:** Pore size of membranes after the application of chemical cleaning agents.

Sample	Average Pore Size (µm)				
	Pristine	1% CA	5% CA	1% NaOH	5% NaOH
PVDF	0.1795	0.1900	0.4419	0.1908	0.2346
PA6	0.3908	0.7658	0.4827	0.5034	0.1834
PAN	0.4401	1.1612	0.5965	0.4265	0.3856

**Table 9:** Contact angle of membranes after applying chemical agents.

Cleaning Solutions	Without Surfactant	With Triton	With SDS
1 wt.% NaOH	77.52 (±2.15)	76.16 (±1.54)	83.72 (±0.77)
5 wt.% NaOH	0	0	86.30 (±0.86)
1 wt.% CA	78.82 (±2.74)	67.24 (±2.50)	78.34 (±0.74)
5 wt.% CA	80.90 (±2.45)	74.25 (±1.25)	103.86 (±2.36)

## Data Availability

Not applicable.
